# Retraction: The silencing of long non-coding RNA ANRIL suppresses invasion, and promotes apoptosis of retinoblastoma cells through ATM-E2F1 signaling pathway

**DOI:** 10.1042/BSR-20180558_RET

**Published:** 2021-09-13

**Keywords:** ATM-E2F1 signaling pathway, Cell apoptosis, Long-non coding RNA ANRIL, Negative feedback regulation, Retinoblastoma

This Retraction follows an Expression of Concern relating to this article previously published by Portland Press.

This article is being retracted from *Bioscience Reports* at the request of the Editor-in-Chief and the Editorial Board following receipt of a notification from a reader, alerting the Editorial Board to duplicated data in Figure 4A which appears in another publication by different authors.

The authors have been contacted in regards to the Retraction, and have not responded to the Journal's queries and the concerns raised. Given the extent of the issues raised, the Editorial Board stand by the decision to retract the article.

